# Distribution épidémiologique de l'infection à VIH chez les femmes enceintes dans les dix régions du Cameroun et implications stratégiques pour les programmes de prévention

**DOI:** 10.11604/pamj.2015.20.79.4216

**Published:** 2015-01-29

**Authors:** Serge-Clotaire Billong, Joseph Fokam, Edson-Joan Billong, Georges Nguefack-Tsague, Marie-Josée Essi, Raoul Fodjo, Samuel-Martin Sosso, Armelle Gomba, Joseph Mosoko-Jembia, Gabriel Loni-Ekali, Vittorio Colizzi, Anne-Cécile Zoung-Kani Bissek, Francisca Monebenimp, Jean-Bosco Elat Nfetam

**Affiliations:** 1 Groupe de Travail National pour la Surveillance et la Prévention de la Pharmacorésistance du VIH (HIVDR-WG), Ministère de la Santé Publique, Yaoundé, Cameroun; 2 Faculté de Médecine et des Sciences Biomédicales (FMSB), Université de Yaoundé 1, Yaoundé, Cameroun; 3 Groupe Technique Central (GTC), Comité National de Lutte contre le SIDA (CNLS), Ministère de la Santé Publique, Yaoundé, Cameroun; 4 Centre International de Référence Chantal BIYA (CIRCB) pour la Recherche sur la Prévention et la Prise en Charge du VIH/SIDA, Yaoundé, Cameroun; 5 Faculté de Médecine, Université Faculté d'Antanarivo, Antanarivo, Madagascar; 6 Centers for Disease Control and Prevention (CDC), Division of Global HIV/AIDS, Bureau-Cameroun; 7 Université de Rome Tor Vergata, et UNESCO Multidisciplinary Biotechnology Board, Rome, Italie; 8 Division de la Recherche Opérationnelle en Santé, Ministère de la Santé Publique, Yaoundé, Cameroun

**Keywords:** VIH, femmes enceintes, Cameroun, HIV, pregnant women, Cameroon

## Abstract

**Introduction:**

Le Cameroun se situe dans un contexte d’épidémie généralisée du VIH. La sous-population des femmes enceintes, facilement accessible au sein de la population générale, représente une cible probante pour mener la surveillance du VIH et estimer l’évolution épidémiologique. L'objectif de notre étude était d’évaluer la distribution épidémiologique du VIH chez les femmes enceintes.

**Méthodes:**

Étude transversale menée en 2012 chez 6521 femmes enceintes (49,3% âgées de 15-24 ans) en première consultation prénatale (CPN1) dans 60 sites des 10 régions Camerounaises. L'algorithme en série a été utilisé pour le sérodiagnostic du VIH.

**Résultats:**

La prévalence du VIH était de 7,8% (508/6521), avec une différence non significative (p = 0,297) entre milieu rural (7,4%) et milieu urbain (8,1%). En zone rurale, cette prévalence variait de 0,7% à l'Extrême-Nord à 11,8% au Sud. Cependant, en zone urbaine elle variait de 4% à l'Ouest à 11,1% au Sud-Ouest. Suivant l’âge, la prévalence était plus élevée (11,3%) chez les femmes de 35-39 ans. Suivant le niveau de scolarisation, la prévalence du VIH était plus faible (4,4%) chez celles non-scolarisées, et plus élevée (9,3%) chez celles ayant un niveau primaire. Selon la profession, l'infection était plus élevée chez les coiffeuses (15,5%), secrétaires (14,8%), commerçantes (12,9%) et institutrices/enseignantes (10,8%).

**Conclusion:**

La prévalence du VIH reste élevée chez les femmes enceintes au Cameroun, sans distinction entre milieux rural et urbain. Les stratégies de prévention devraient s'orienter préférentiellement chez les femmes enceintes âgées, celles du niveau d'instruction primaire, et celles du secteur des petites et moyennes entreprises.

## Introduction

La population Camerounaise était estimée au total à 19 400 000 habitants en 2010, pour une répartition d'environ 52% de femmes [1]. De même, avec une prévalence du VIH à 4,3% dans la population générale, le pays reste en contexte d’épidémie dite généralisée, avec presqu'une double féminisation de l'infection [2]. Dans ce contexte les enquêtes démographiques et de santé (EDS) sont idéales pour surveiller les mouvements de l’épidémie. Compte tenu de leur coût très élevé, il est fortement recommandé par l'Organisation Mondiale de la Santé (OMS) de réaliser ces EDS tous les 5ansdans l'optique d’évaluer l'impact des programmes de prévention et de prise en charge de l'infection à VIH sur la dynamique de l’épidémie [3]. Toutefois, entre deux enquêtes EDS, la tendance de la prévalence du VIH au sein de la population générale s'effectue sur le terrain par la mise en ‘uvre des études de surveillance sentinelle auprès des sous-populations, dont les femmes enceintes, les travailleuses de sexe, les hommes en tenue, les camionneurs de longues distance, les hommes ayant les rapports sexuels avec des hommes (HSH), etc. Parmi celles-ci, les femmes enceintes constituent la cible privilégiée dans notre contexte [3]. A la suite de l'EDS réalisée en 2012 au Cameroun [2], il est donc primordial de continuer la surveillance épidémiologique nationale de l’épidémie à VIH par une étude de surveillance sérologique du VIH auprès des femmes enceintes en première consultation prénatale, celles-ci représentant non seulement une des cibles prioritaires, mais aussi la plus facilement accessible sur le plan programmatique [3]. Il est à noter que, la surveillance épidémiologique du VIH, du sida et des IST a pour but d'orienter les activités de lutte contre cette épidémie et les infections associées, en fournissant des informations stratégiques de qualité sur l'ampleur et sur la distribution nationale de l'infection à VIH. Les résultats de la surveillance sont donc essentiels pour l'estimation de la tendance épidémiologique nationale, l’élaboration de meilleures stratégies de planification, de mise en ‘uvre, de suivi et d’évaluation des programmes de prévention de l’épidémie, partant des groupes cibles vers une application opérationnelle des évidences probantes au sein de la population générales [3].

Par ailleurs le Cameroun dispose actuellement d'un Plan Stratégique National de lutte contre le VIH, le SIDA et les IST jusqu'en 2015 [4], et le programme national de lutte contre le SIDA s'est doté d'un plan d’élimination virtuelle de la transmission du VIH de la mère à l'enfant à l'horizon 2015 [5]. Le suivi et l’évaluation de ces stratégies en cours requièrent entre autre la maîtrise de l'incidence et de la prévalence de l'infection à VIH dans ces sous-groupes ou populations prioritaires. En effet, sur un volet programmatique et opérationnel, la connaissance de cette prévalence servirait à déterminer les quantifications et la gestion des approvisionnements et des stocks d'ARV, de même qu’à l’évaluation des intrants et l’élaboration des requêtes de financement sur des bases crédibles/vérifiables à divers agences de financement de la lutte contre le VIH/SIDA [4, 5]. De plus, des études récentes faites sur des groupes cibles démontrent qu'au Cameroun, la baisse de la prévalence générale ne s'accompagne pas systématiquement d'une baisse dans toutes les sous-populations (42% de prévalence du VIH chez les HSH à Yaoundé) [6]; l’épidémie pouvant donc se loger dans tels sous-groupes (HSH et vraisemblablement chez les travailleurs de sexe) qui deviennent ainsi des moteurs de l’épidémie. L'objectif de la présente étude était d’évaluer la prévalence du VIH dans les sites sentinelles de l'infection à VIH au Cameroun. Spécifiquement, il était question d'apprécier la variabilité de l'infection à VIH en fonction des dix régions géographiques du pays (y compris les comparaisons entre milieux urbains et ruraux), d’évaluer la distribution épidémiologique en fonction de l’âge, du statut professionnel et du niveau d’éducation; sous hypothèse selon laquelle la prévalence de l'infection à VIH évaluée en 2009 à 7% a baissé parallèlement à celle dans la population générale qui est passée de 5,5% en 2004 (EDS) à 5,1% en 2009 (CNLS/ EPP Spectrum) puis à 4,3% en 2011 (EDS).

## Méthodes

**Cadre Conceptuel de l'Etude:** une étude prospective et transversale, à visée analytique, a été menée dans les dix régions du Cameroun de Janvier à Décembre en 2012, et précisément sur l'ensemble des 20 sites et 60 points de collecte habituels de surveillance sentinelle du VIH au Cameroun. Suivant un échantillonnage par convenance (i.e. choix de façon raisonnée et non-aléatoire tel que requis par les critères de sélection dans toute surveillance sentinelle), les sites de surveillance sentinelle existants ont été choisis avec une représentativité régionale du pays et la localisation géographique (urbaine/rurale). Sur le plan méthodologique, un site était défini comme un ou plusieurs district(s) de santé situé(s) soit en zone urbaine soit en zone rurale, ayant un taux de couverture en consultation prénatale (CPN) d'au moins 60% (seuil requis par l'OMS) et pouvant réunir un ensemble de 300-500 femmes en première CPN (CPN1) pendant la durée de collecte de données. Pour l’éligibilité comme point de collecte, une formation sanitaire devait remplir les critères suivants: (i) Offrir les services de CPN et de prévention de la transmission mère-enfant du VIH (PTME); (ii) Avoir été un point de collecte de surveillance sentinelle du VIH en CPN; (iii) Disposer d'un service de dépistage du VIH et (iv) Disposer des données de PTME des trois mois précédant l'enquête. Notre population d’étude était donc constituée de toutes les femmes enceintes venant pour la première fois en visite de CPN, recrutées consécutivement jusqu’à l'atteinte de la taille de l’échantillon requis (300 ou 500 personnes par site sentinelle); la taille de l’échantillon étant calculée avec un seuil d'incertitude de 5% et selon les critères recommandés par l'ONUSIDA/OMS, [7].

**Figure 1:**
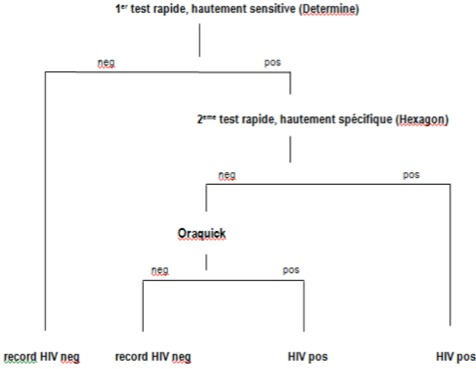
Algorithme national pour le dépistage anonyme et volontaire par tests rapides

**Analyses Biologiques de l'Etude:** le Centre International de Recherche Chantal BIYA (CIRCB) pour la recherche sur la prévention et la prise en charge du VIH/SIDA a été choisi comme laboratoire national de référence (LNR) à la suite d'une série d’évaluations de performance technique(au départ et durant l’étude) pour l'assurance qualité durant l’étude. La collecte des échantillons et données ont été effectuées altération des méthodes de travail dans les sites. Habituellement, le dépistage du VIH était proposé à toutes les femmes enceintes en CPN1, et sur le bulletin d'examen ou le carnet de la femme, le numéro de CPN était noté et la case intitulée ‘VIH’ cochée en cas d'acceptation du test. Après centrifugation/sédimentation du sang total, le sérum/plasma était recueilli dans un cryotube, préalablement étiqueté avec le code puis conservé dans un réfrigérateur entre 0 et 8°C. Ces cryotubes étaient mis dans une cryoboite puis transférés au LNR pour le diagnostic sérologique du VIH, avec le bordereau de transmission, respectant les normes universelles de transfert de matériels biologiques [8]. L'algorithme en série a été utilisé pour le dépistage du VIH, avec comme premier test **« Determine HIV1/2»** (Abbott, Minato-ku-Tokyo,Japan), deuxième test « Hexagon »(Kora Healthcare, Ireland, United Kingdom), et pour des cas indéterminés un troisième test **« Oraquick »** (OraSure Technologies, Inc, Bethlehem, Pennsylvania) [9]. Cet algorithme est résumé en Figure 1.

**Assurance Qualité et Interprétation des résultats**: afin d'assurer la qualité de la mise en ‘uvre de cette étude, tous les acteurs ont été formés sur les taches à accomplir, parmi lesquelles: la méthode du remplissage des registres anténatals et des formulaires de surveillance sentinelle; les procédures opératoires standards pour les phases pré-analytique, analytique et post-analytique des tests de dépistage; le suivi de l’étude, la vérification des données et transfert des échantillons des points de collecte vers le LNR. Le suivi était assuré par des supervisions centrales et régionales, dans l'optique d’évaluer et de renforcer la qualité des données à l'aide d'une application informatique (RDQA: Data Quality Assessment) spécialement conçue à cet effet. Une vérification de la transcription des données des registres vers les fiches de collecte a été effectuée au niveau des points de collecte. Une fois au niveau central, une double-saisie des données a été réalisée afin de minimiser de potentielles erreurs de saisie dans une base de données conçue sous le logiciel CSPro (Census and Survey Processing), version 4.1. Les données ont ensuite été apurées, analysées et archivées. Le laboratoire du CIRCB a été évalué par le Center for Disease Control and prévention (CDC) pour sa sélection comme laboratoire national de référence pour cette enquête. Cette évaluation, menée à l'aide des panels périodiques (i.e. au début puis trois fois durant l'enquête) a permis de s'assurer de la fiabilité des résultats reportés; la concordance exigée de 100% étant régulièrement obtenue dans tous les panels.

**Analyse Statistique des Données:** la séroprévalence (P) du VIH dans les sites de surveillance sentinelle a été calculée par la formule **P = x/n, x** étant le nombre total de femmes enceintes dont le test était positif pour le VIH et n le nombre total de prélèvements testés dans un site donné ou un point de collecte. En multipliant cette proportion par 100%, on a obtenu la prévalence du VIH en pourcentage. Pour le calcul de la prévalence agrégée au niveau régional et au niveau national, deux méthodes de calcul ont été utilisées. La première méthode est celle de la moyenne pondérée des prévalences des sites de chaque région. Le coefficient de pondération étant le nombre de femmes testées dans le site. La seconde méthode est celle de la médiane. Elle est recommandée par le guide de l'OMS sur les recommandations pour les enquêtes sérologiques sentinelles concernant le VIH chez les femmes enceintes et autres groupes [7]. Etant donné que la taille de l’échantillon des femmes enceintes recrutées au niveau des points de collecte variait entre 100 et 200, la séroprévalence de chaque site a été obtenue en regroupant les données sur la prévalence de ses points de collecte.

**Considérations éthiques: n**otre étude a été approuvée par le Comité Ethique Nationale (CNE/2011), et une autorisation administrative a été obtenue au Ministère de la Santé Publique. Suivant le principe de surveillance non corrélée, l'obtention d'un consentement éclairé des femmes enrôlées n’était pas requis, permettant ainsi de minimiser tout biais de sélection lié à l'acceptation du test de dépistage du VIH et d’évaluer le programme en situation réelle dans ses pratiques quotidiennes en routine. Tout de même, la confidentialité a été assurée dans le traitement des données, et suivant les directives nationales en matière de CPN et PTME.

## Résultats

**Profil sociodémographique des femmes enceintes enquêtées**: un total de 6521 femmes enceintes a été enrôlé dans l'enquête sur l’étendue nationale durant la période de l’étude, dont 2784 en milieu rural et 3737 en milieu urbain. Le Tableau 1 présente la répartition détaillée des femmes enceintes enquêtées par région selon la localisation du site, chaque région comportant un site urbain et un site rural. Sur la base de l’échantillonnage prévu à la base, le taux de couverture de l’échantillon a été de 93,2% au niveau national, sans différence significative (p= 0,476) entre le milieu rural (92,8%) et le milieu urbain (93,4%). Le gap observé (6,8%) a été grandement du au faible taux de couverture de l’échantillonnage dans la région du Sud, faute d'atteinte de la taille requise de l’échantillon en raison du rejet de certains prélèvements ou fiches parle dispositif d'assurance qualité. En fonction de l’âge, près de la moitié des femmes enceintes enquêtées étaient jeunes (49,3% âgées entre 15-24 ans), avec 75,0% âgées de moins de 30 ans. Suivant le niveau de scolarisation, la grande majorité (83,6%) des femmes enceintes enquêtées ont été à l’école, parmi lesquelles 75,3% avaient atteint les niveaux primaire ou secondaire, et 8,3% le niveau supérieur. Le taux de scolarisation variait en fonction de la zone de localisation du site (les femmes en zone urbaine étaient plus instruites que celles en zone rural). Sur le plan professionnel, la moitié de ces femmes (49,7%) étaient des menagères, 14,2% des étudiantes ou élèves, et environ 1/3 (33%) étaient des employées.

**Tableau 1:** Répartition des femmes enceintes enquêtées par région et selon la localisation

	RURAL		URBAIN		ENSEMBLE	
REGION	Nombre de femmes enceintes enquêtées	Taux de couverture de l’échantillon en%	Nombre de femmes enceintes enquêtées	Taux de couverture de l’échantillon en%	Nombre de femmes enceintes enquêtées	Taux de couverture de l’échantillon en%
Adamaoua	301	100,33%	412	103,00%	713	101,86%
Centre	244	81,33%	317	79,25%	561	80,14%
Est	327	109,00%	303	75,75%	630	90,00%
Extrême-Nord	295	98,33%	383	95,75%	678	96,86%
Littoral	271	90,33%	403	100,75%	674	96,29%
Nord	299	99,67%	428	107,00%	727	103,86%
Nord-Ouest	302	100,67%	392	98,00%	694	99,14%
Ouest	294	98,00%	398	99,50%	692	98,86%
Sud	152	50,67%	321	80,25%	473	67,57%
Sud-ouest	299	99,67%	380	95,00%	679	97,00%
Total	2784	92,80%	3737	93,43%	6521	93,16%

**Figure 2:**
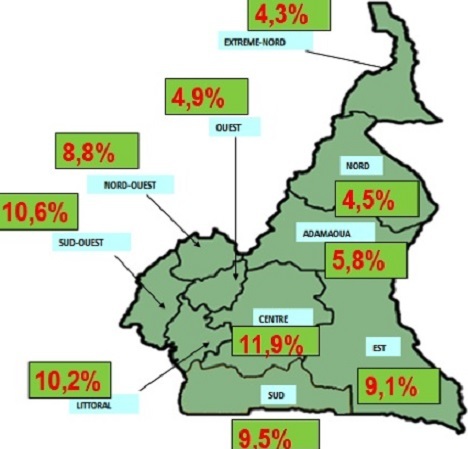
Prévalence du VIH chez les femmes enceintes en CPN 1 par région en 2012

**Distribution épidémiologique du VIH dans la population générale de l’étude**: apres confirmation du statut de l'infection de tous les échantillons collectés, le test du VIH s'est révélé positif pour 508 femmes enceintes parmi les 6521 femmes testées, ce qui correspond à une prévalence nationale du VIH de 7,8% chez les femmes enceintes, avec une distribution régionale variée (Figure 2). La distribution épidémiologique variait de 7,4% en milieu rural à 8,1% en milieu urbain, la différence n’étant pas statistiquement significative (p = 0,297). Ainsi, au seuil de risque d'erreur de 5%, l'hypothèse selon laquelle il existe une différence statistique entre la prévalence en milieu urbain et celle en milieu rural a été rejetée. De plus, dans la zone rurale, la distribution épidémiologique chez les femmes enceintes enquêtées variait de 0,7% à l'Extrême Nord à 11,8% au Sud; alors que dans la zone urbaine cette prévalence variait de 4% à l'Ouest à 11,1% au Sud-Ouest. Le Tableau 2 présente la prévalence du VIH chez les femmes enceintes enquêtées par région et selon la zone de localisation rurale ou urbaine du site au Cameroun.

**Tableau 2:** Prévalences régionales du VIH chez les femmes enceintes enquêtées

REGIONS	RURAL		URBAIN		ENSEMBLE		
	Nombre de femmes enceintes enquêtées	Prévalence du VIH d'après le LNR	Nombre de femmes enceintes enquêtées	Prévalence du VIH d'après le LNR	Nombre de femmes enceintes enquêtées	Prévalence du VIH (moyenne pondérée)	Prévalence du VIH (médiane)
Adamaoua	301	6,98%	412	4,85%	713	5,75%	5,92%
Centre	244	9,43%	317	13,88%	561	11,94%	11,63%
Est	327	10,70%	303	7,26%	630	9,05%	8,98%
Extrême-Nord	295	0,68%	383	7,05%	678	4,28%	3,86%
Littoral	271	9,59%	403	10,67%	674	10,24%	10,17%
Nord	299	3,01%	428	5,61%	727	4,54%	4,30%
Nord-Ouest	302	7,95%	392	9,44%	694	8,79%	8,69%
Ouest	294	6,12%	398	4,02%	692	4,91%	5,07%
Sud	152	11,84%	321	8,41%	473	9,51%	10,13%
Sud-ouest	299	10,03%	380	11,05%	679	10,60%	10,54%
Total par la moyenne pondérée	2784	7,40%	3737	8,08%	6521	7,79%	
Total par la médiane	2784	8,67%	3737	7,84%	6521	8,18%	

**Distribution épidémiologique du VIH en fonction de l’âge et de la zone de localisation du site:** en fonction de la tranche d’âge, les femmes de 35-39 ans ont la prévalence la plus élevée (11,3%), contre celles âgées de plus de 45 ans ou l'on n'a enregistré aucun cas de séropositivité au VIH (Tableau 3). La distribution épidémiologique du VIH par âge diffère selon la zone de localisation du site, le milieu urbain ayant une prévalence augmentant rapidement avec l’âge (passant de 3,0% chez les 15-19 ans pour atteindre un maximum de 12,3% chez les 35-39 ans, avec p=: la distribution épidémiologique en fonction du niveau d'instruction démontre que la prévalence du VIH était plus faible (4,4%) chez les femmes enceintes non scolarisées, aussi bien en milieu rural qu'en milieu urbain. Par ailleurs, la prévalence la plus élevée (9,3%) a été reportée chez les femmes enceintes ayant atteint le niveau d'instruction primaire. Ainsi, l'absence absolue ou dans une certaine mesure le dépassement du niveau d'instruction de base était probablement associée, à des degrés variables, à une éventuelle protection contre l'infection à VIH. Les résultats détaillés sont reportés dans le Tableau 4.

**Tableau 3:** Prévalence du VIH chez les femmes enceintes en fonction de l’âge

TRANCHE D'AGE	LOCALISATION DU SITE					
	RURAL		URBAIN		TOTAL	
	Nombre de femmes enceintes enquêtées	prévalence du VIH	Nombre de femmes enceintes enquêtées	prévalence du VIH	Nombre de femmes enceintes enquêtées	prévalence du VIH
15 - 24 ans	1459	4,87%	1758	5,63%	3217	5,28%
15 - 19 ans	584	3,94%	609	2,96%	1193	3,44%
20 - 24 ans	875	5,49%	1149	7,05%	2024	6,37%
25 - 49 ans	1325	10,19%	1979	10,26%	3304	10,23%
25 - 29 ans	673	11,00%	1003	9,07%	1676	9,84%
30 - 34 ans	406	9,85%	635	11,65%	1041	10,95%
35 - 39 ans	172	9,88%	269	12,27%	441	11,34%
40 - 44 ans	69	5,80%	68	7,35%	137	6,57%
45 - 49 ans	5	0,00%	4	0,00%	9	0,00%
Total	2784	7,40%	3737	8,08%	6521	7,79%

**Tableau 4:** Prévalence du VIH chez les femmes enceintes suivant le niveau d'instruction et la localisation

NIVEAU D'INSTRUCTION	LOCALISATION DU SITE					
	RURAL		URBAIN		TOTAL	
Nombre de femmes enceintes enquêtées	prévalence du VIH	Nombre de femmes enceintes enquêtées	prévalence du VIH	Nombre de femmes enceintes enquêtées	prévalence du VIH
Jamais fréquenté	605	3,80%	466	5,15%	1071	4,39%
Primaire	1088	8,92%	1231	9,59%	2319	9,27%
Secondaire	995	7,74%	1597	8,08%	2592	7,95%
Supérieur	96	9,38%	443	7,00%	539	7,42%
Total	2784	7,40%	3737	8,08%	6521	7,79%

**Distribution épidémiologique du VIH suivant la profession et la localisation**: suivant le statut professionnel, la prévalence du VIH chez les femmes enceintes est plus élevée chez les coiffeuses (15,5%), les secrétaires (14,8%), les commerçantes (12,9%) et les institutrices/enseignantes (10,8%); une tendance maintenue aussi bien en milieu urbain qu'en milieu rural. Toutefois, les données statistiques dans ces différents effectifs de femmes suscitées ne permettaient pas de tirer une conclusion définitive par rapport à des prévalences plus élevées chez les femmes appartenant à ces sous-groupes de métiers. Le Tableau 5 montre la prévalence de l'infection en fonction du statut professionnel et la zone de localisation du site.

**Tableau 5:** Prévalence du VIH chez les femmes enceintes suivant la profession et la localisation du site

	LOCALISATION DU SITE					
	RURAL		URBAIN		TOTAL	
STATUT PROFESSIONNEL	Nombre de femmes enceintes enquêtées	prévalence du VIH	Nombre de femmes enceintes enquêtées	prévalence du VIH	Nombre de femmes enceintes enquêtées	prévalence du VIH
Commerçante	194	13,40%	366	12,57%	560	12,86%
Personnel médical	21	*	67	4,48%	88	3,41%
Femme en tenue	4	*	12	*	16	*
Cultivatrice/Agriculture	351	9,97%	125	8,00%	476	9,45%
Ménagère	1567	6,25%	1676	7,16%	3243	6,72%
Etudiante/Elève	313	4,15%	614	6,35%	927	5,61%
Sans emploi	77	6,49%	120	8,33%	197	7,61%
Institutrice/Enseignante	63	12,70%	169	10,06%	232	10,78%
Couturière	84	2,38%	246	7,32%	330	606%
Secrétaire	12	*	49	12,24%	61	14,75%
Coiffeuse	52	13,46%	135	16,30%	187	15,51%
Autres	46	19,57%	158	6,96%	204	9,80%
Total	2784	7,40%	3737	8,08%	6521	7,79%

* **Légende**: Valeur est basée sur moins de 30 échantillons et donc supprimée.

## Discussion

Le but de cette étude était de fournir aux gestionnaires des programmes de prévention de la transmission Mère-enfant du VIH, une information stratégique, qui en même temps est le principal intrant des modélisations mathématiques par logiciels tels que EPP et SPPECTRUM, permettant de déterminer les projections de l’épidémie à VIH (utiles pour définir les besoins en ARV, les nouvelles infections, et l'efficacité des stratégies préventives actuelles) [3, 10]. L'objectif de la présente étude était d’évaluer la prévalence du VIH chez les femmes enceintes au cours de l'année 2012 dans le cadre de la surveillance sentinelle de l'infection à VIH. La prévalence nationale du VIH a effectivement été estimée à 7,8% sur l'ensemble de la population étudiée en 2012, partant de 7,6% en 2009 sur les mêmes sites, traduisant une épidémiologique relativement stable entre 2009 et 2012. Plus spécifiquement, l’épidémiologique de 2009-2012 en milieu rural allait de 6,59% à 7,4%, et en milieu urbain de 8,21% à 8,1%. Toutefois, l’épidémie est en augmentation dans près de la moitié des sites (9/20), dont 5 ruraux (Centre, Est, Littoral, Ouest, et Sud) et 4 urbains (Centre, Extrême-Nord, Littoral, et Nord-Ouest). La plus basse prévalence variait de 4,3% en 2009 à 3,86% en 2012 toujours à l'Extrême-Nord; les plus élevées en 2009 se situaient au Sud-Ouest, à l'Est, au Sud ( 11,94%, 9,27%, 9,0% respectivement), puis en 2012 dans les régions du Sud-Ouest (10,54%), Littoral (10,17%) et Sud (10,17%) [11]. Ainsi, les régions du Sud-Ouest et du Sud restent les plus fortement touchées par l’épidémie du VIH chez les femmes enceintes, en accord avec les résultats de la récente EDS de 2011 et soulignant ainsi des interventions prioritaires en matière de prévention dans ces régions nationales [2]. De plus, l'importante variation épidémiologique (en augmentation ou en réduction) dans les sites sentinelles indiquerait le comportement de l’épidémie du VIH dans la population générale et celle des femmes en particulier [11]. Les EDS 2004-2011 montrent pourtant une réduction en population générale [2, 12]. Ainsi, malgré la baisse significative entre 2004-2011, l’épidémie est plutôt restée stable ces dernières années [11]. Cette stabilité nécessite d’être investiguée, étant donné qu'il pourrait s'agir en effet d'un équilibre entre une mortalité élevée et une incidence élevée, traduisant dans de telle contexte un échec des stratégies actuelles de prévention et de prise en charge. En plus, elle a augmenté dans les sites urbains (Yaoundé et Douala) et ruraux des Régions du Centre et du Littoral en conformité avec EDS [2, 12]. Par ailleurs, bien que globalement basse comparativement aux autres tranches étudiées, la prévalence du VIH chez les 15 ‘ 18 ans est très élevée car, il s'agit en général de nouvelles infections orchestrées au sein de la communauté [2, 11], [12]. Toutefois, le pourcentage de femmes enceintes de la tranche 15-24 ans infectées au VIH est plus important en milieu urbain (5,6%) qu'en milieu rural (4,9%). Ces résultats indiqueraient de ce fait une recrudescence des nouvelles infections seraient, plus importantes en zone rurale qu'en zone urbaine; des tendances similaires étant observées avec les résultats observés dans la population générale (EDS) [2] et dans d'autres pays à ressources limitées, et particulièrement en Afrique sub-Saharienne présentant des caractéristiques sociodémographiques comparables (i.e. Sénégal, République Démocratique du Congo, Zimbabwe) [13-19].

Dans le domaine de la santé en général, l’éducation est un facteur favorable au décodage des messages de communication émis pour les changements de comportement ou pour la mobilisation et le marketing social en faveur de la lutte contre les maladies [4]. Le résultat montre paradoxalement que la prévalence du VIH est plus faible (4,4%) chez les femmes enceintes enquêtées n'ayant jamais été scolarisées aussi bien en milieu rural qu'en milieu urbain, résultats similaires observés durant l’étude menée en 2009, ce qui traduit de ce fait une probable mortalité élevée ou une plausible faible fréquentation de plusieurs cas de femmes infectées dans ce groupe, au lieu d'une éventuelle protection telle que semble montrer nos résultats. Les explorations explicatives ont été observées dans des d'autres pays en développement [13-20], sonnant ainsi une alerte importante à prendre en compte par les gestionnaires des programmes de communication étant la prévalence la plus élevée (9,3%) rencontrée chez les femmes enceintes ayant atteint mais s’étant limitées à l’école primaire. La prévalence du VIH serait importante chez les femmes enceintes exerçant tout petit métier, bien que la faible taille d’échantillons dans ces groupes limite l'implication opérationnelle de cette observation qui demeure tout de même conforme aux résultats obtenus en 2009 dans ces mêmes sites au Cameroun [11]. Une autre potentielle limite de cette étude est que l’échantillon reste peu représentatif de la population étudiée, étant donné qu'il s'agit d'une étude épidémiologique dans les sites sentinelles qui ne peut être inférée a la population générale qu'après des ajustements statistiques (i.e. EPP/Spectrum) pour une extrapolation nationale, afin que l'interprétation de ces résultats gagne toute sa valeur épidémiologique [3, 21], [22]. En plus des études menées précédemment dans le pays [23-25], cette population de femmes enceintes servirait aussi d’échantillonnage pour une évaluation rationnelle du seuil actuel des résistances transmises du VIH-1 au Cameroun, suivant les critères recommandés par l'OMS [26].

## Conclusion

La prévalence nationale de l'infection à VIH est restée globalement stable chez les femmes enceintes ces trois dernières années, ce qui suppose une baisse de performances, car dans l'intervalle des 06 dernières années elle a en moyenne baissé dans la population générale. Un renforcement des programmes de prévention, particulièrement en milieu jeune, en faible scolarisation (niveau primaire) et dans le secteurs des petits métiers; les Régions du Sud-Ouest, du Littoral et du Sud méritent une attention particulière. Bien que complexes, des études d'incidence et de mortalité liées à l'infection à VIH permettraient une meilleure compréhension des tendances nationales de l’épidémie à VIH au Cameroun et dans tout pays partageant de telles similarités socioculturelles et économiques.
